# Diagnostic value of C-reactive protein-albumin-lymphocyte index in breast cancer cases

**DOI:** 10.3389/fonc.2026.1719184

**Published:** 2026-05-07

**Authors:** Mustafa Cem Algin, Murat Özgür Kılıç, Mustafa Salış, Ozlem Arik, Orhan Kalayci, Bahattin Erdogan, Ali Uncu, Ruhin Abishov, Ulku Busra Bayram

**Affiliations:** 1 Ministry of Health Eskisehir City Hospital, Eskisehir, Türkiye; 2 Necmettin Erbakan University, Faculty of Medicine, Department of Biostatistics and Medical Informatics, Konya, Türkiye

**Keywords:** breast cancer, CALLY index, diagnostic prognostic biomarker, inflammation, machine learning, staging

## Abstract

**Background:**

The most frequently diagnosed malignancy among women worldwide is breast cancer. Inflammation, nutritional status, and immune competence are increasingly recognized as major determinants of tumor progression and prognosis in cancer cases. The C-reactive protein–albumin–lymphocyte (CALLY) index, a composite biomarker reflecting these parameters, has shown promise in studies of various tumors. However, research on the CALLY index in breast cancer remains limited in the literature.

**Objective:**

In this study, we evaluated the diagnostic significance and staging performance of the CALLY index in patients with breast carcinoma.

**Method:**

A total of 303 female patients diagnosed with breast cancer between November 2018 and December 2024 were retrospectively analyzed. Pre-treatment serum CRP, albumin, and lymphocyte counts were used to calculate the CALLY index (Albumin [g/dL] × Lymphocyte count [/µL])/(CRP [mg/dL] × 10^4)^. Patients were classified into early-stage (Stage I-II) and advanced-stage (Stage III-IV) groups. Group comparisons were performed using Mann-Whitney U and Kruskal-Wallis tests. ROC analysis and supervised machine learning algorithms were used to evaluate the discriminative performance of the CALLY index and CA-15-3.

**Results:**

The mean CALLY index was significantly higher in early-stage patients compared to those with advanced-stage disease (p < 0.001). The index demonstrated excellent discriminative ability for staging, with an optimal cut-off value of 0.693 (AUC: 1.00; sensitivity: 100%; specificity: 100%). In multivariate analysis, the CALLY index outperformed CA 15–3 in predicting tumor stage. Machine learning classification confirmed that the CALLY index was the most important variable for stage prediction, and the neural network model achieved an accuracy of 84.9% on the test set.

**Conclusion:**

The CALLY index is one of the non-invasive and easily accessible biomarkers that can distinguish between early and advanced breast cancer by integrating inflammatory, nutrition and immune parameters. Furthermore, the CALLY index holds significant potential for pre-treatment prognostic stratification of breast cancer in clinical practice.

## Introduction

The most frequently diagnosed malignancy among women worldwide is breast cancer. Breast cancer is one of the leading causes of cancer-related deaths. When the report prepared by GLOBOCAN 2023 is examined, it is seen that breast cancer constitutes 11.7% of cancer cases worldwide. Breast cancer has an incidence trend in developing countries and high-income countries (1).

Despite significant advances in multimodal treatment and early diagnosis strategies in breast cancer, it has been determined that prognostic stratification continues to be a complex challenge due to the heterogeneous biological behavior of breast tumors. There is an increasing demand for affordable, reliable and easily accessible biomarkers that reflect tumor biology, including tumor grade, anatomical staging, oncogene expression, hormone receptor status and gene expression, and help in diagnosis and prognosis prediction (2–4).

Currently widely used, CA 15–3 is a noninvasive, readily available, and cost-effective tumor marker for follow-up prediction of breast cancer (5). Rhu et al. demonstrated that elevated CA15–3 levels have a prognostic impact in patients with early breast cancer and normal serum CA15–3 levels (6).

Nutritional status, inflammatory response and host immune competence play a critical role in cancer progression and patient outcomes (7, 8). Immune cell markers such as inflammatory markers (C-reactive protein (CRP)), lymphocyte count and nutritional indicators such as serum albumin are associated with survival outcomes in different malignancies, including breast cancer. These single-parameter biomarkers are inadequate in capturing the multifactorial nature of cancer-associated systemic disorders (9, 10).

Composite indices integrating more than one biological dimension have been developed to examine this situation. One of these developed composite indices is the C-reactive protein-albumin-lymphocyte (CALLY) index. The CALLY Index was first proposed by Lida et al. for patients with hepatocellular carcinoma. It was later determined that it was validated in many malignancies, including esophageal, stomach, oral cavity and pancreatic cancers (11–20). This biomarker outperforms traditional biomarkers such as platelet-lymphocyte ratio, neutrophil-lymphocyte ratio, and modified Glasgow Prognostic Score (8, 21). Lower CALLY index has been associated with higher postoperative complications and worse prognosis rates in kidney, pancreas, and esophageal cancer cohorts (8, 12, 13, 22, 23).

The prognostic benefit of the CALLY index is supported by evidence, especially in breast cancer. It was found that a CALLY index below 2.285 was independently associated with worse overall and disease-free survival in patients undergoing breast cancer surgery (24).

Studies have shown that combining nutrition, inflammation, and immunity into a single measurement provides superior prognostic classification compared to traditional indicators. Given the simplicity and reproducibility of the CALLY Index compared to routine laboratory parameters, it offers a practical tool for the global oncology community. Conversely, CA15–3 is a serum tumor marker for breast cancer that is still widely used in clinical practice. Studies have shown that elevated CA15–3 levels are a prognostic indicator (6). This study aimed to evaluate the diagnostic value and staging performance of the CALLY Index in patients diagnosed and undergoing surgery for breast carcinoma by comparing it with CA 15–3 level.

## Materials and methods

### Study design and population

This retrospective observational cohort study was conducted to observe the CALLY index diagnostic value and staging performance in patients with breast cancer. 303 female patients aged between 21–70 years who were diagnosed with invasive breast carcinoma in Eskisehir City Hospital General Surgery Clinic between November 2018 and December 2024 were included in the study. All cases here were confirmed by histopathological examination, and staging was performed according to the American Joint Committee on Cancer (AJCC) 8th Edition TNM classification (2). The study protocol was reviewed and approved by the Scientific Research Ethics Committee of Eskisehir City Hospital. The study was conducted in accordance with the Declaration of Helsinki. Since all data in the study were anonymized, informed consent was not obtained.

### Eligibility criteria

#### Inclusion criteria

Patients who had pre-treatment laboratory data such as serum CRP, albumin, lymphocyte count and CA 15-3, whose histologically confirmed diagnosis of primary breast cancer and who had not received any treatment related to other organ cancers before breast cancer treatment were included in the study.

#### Exclusion criteria

Patients with missing laboratory or staging data, patients with hematological malignancy, active infection or autoimmune disease during the diagnosis process, and patients using immunosuppressive or anti-inflammatory drugs were excluded from the study.

### Data collection

For each patient participating in the study, laboratory parameters three weeks before surgery were extracted from the hospital database. These parameters included serum albumin (g/dL), CRP (mg/dL), lymphocyte count (cells/μL), and CA 15–3 levels. Pathological features and staging data were obtained from surgical pathology reports. The CALLY index was calculated using the following formula;

CALLY index = (Albumin [g/dL] × Lymphocyte count [/μL])/(CRP [mg/dL] × 10^4^).

The CALL index formula was designed to reflect nutritional status, systemic inflammation, and immune competence in a single numerical value. This design was adapted from previous literature.

In the study, the effect of the CALLY index on early-stage (Stage I-II) and advanced-stage (III-IV) was determined by comparatively evaluating the diagnostic performance in distinguishing breast cancer and the levels of the CALLY index and CA 15–3 between clinical stages. Here, ROC analysis was used for each marker. The normalized variable significance order was evaluated by determining the diagnostic criteria and machine learning-based staging classification.

### Statistical analysis

Statistical analyzes were conducted using SPSS 26 and MedCalc 14.0. The distribution of continuous variables was assessed with the Shapiro–Wilk–Francia and Kolmogorov–Smirnov tests, and homogeneity of variances with the Levene test. For comparisons between two groups, the Mann–Whitney U test was applied, whereas the Kruskal–Wallis H test with Dunn’s *post hoc* was used for comparisons among more than two groups. Monte Carlo simulations were performed to confirm significance. Supervised machine learning algorithms including discriminant analysis, logistic regression, decision tree, k-nearest neighbor, random forest, support vector machine, Naive Bayes classification, and multilayer perceptron (MLP) neural network were employed. The dataset was randomly divided into stratified training (70%) and test (30%) subsets, maintaining class proportions across both subsets. Preprocessing steps were fitted only on the training set and then applied to the test set to avoid data leakage. In the MLP model, the sigmoid function was used as the hidden layer activation function, exponential/sigmoid functions were used at the output layer, and mini-batch gradient descent was implemented as the optimization algorithm. Hyperparameters (number of hidden layer neurons, learning rate, batch size, and regularization coefficient) were determined through preliminary experiments. Model performance was evaluated using accuracy, sensitivity, specificity, positive predictive value (PPV), negative predictive value (NPV), F1-score, Matthews correlation coefficient (MCC), and the area under the receiver operating characteristic curve (ROC-AUC). ROC Analysis and Cut-off Values: ROC analyzes were performed to distinguish early (I–II) from advanced (III–IV) stages. AUC values with 95% confidence intervals were reported. Cut-off values were determined using the Youden index; however, these thresholds were not re-tested in an independent validation cohort, which represents a methodological limitation. In addition, PPV and NPV values are specific to the prevalence in this manuscript sample and may vary in external populations.

Since partial imbalance existed between stages, stratified splitting was applied to maintain class proportions in both training and test sets. Furthermore, in the MLP model, class weights were incorporated into the loss function to penalize misclassifications of the minority class more heavily, thereby improving sensitivity. Alternative approaches such as SMOTE or focal loss were not implemented in this study.

Continuous variables are presented as mean ± SD, median (min–max, Q1–Q3), while categorical variables are expressed as n (%). A p-value <0.05 was considered statistically significant with 95% confidence intervals.

## Results

Data from 303 women were analyzed in the study. Stage II and Stage IV breast cancer were diagnosed in 100 patients (33.0%) and 85 patients (28.1%), respectively. The proportion of early-stage cases (Stage I-II, n = 162) was 53.5%, while advanced-stage cases (Stage III-IV, n = 141) accounted for 46.5%. The mean CRP level was 2.68 ± 3.33 mg/dL (min: 0.15, max: 14.5), albumin was 3.67 ± 1.36 g/dL (min: 1.0, max: 6.6), mean lymphocyte level was 2.53 ± 1.00 X 10³/μL (min: 1.1, max: 4.5) and the mean CALLY index was 1.78 ± 1.64 (min: 0.008, max: 6.88). The mean CA 15–3 level was 53.18 ± 53.06 U/mL (min: 3, max: 407) (**Table 1**).

**Table 1:** Distribution of clinical stages and summary of inflammatory and tumor markers.

Stages / Inflammatory and Tumor Markers	n	%
Stage			
I	62	20.5%
II	100	33.0%
III	56	18.5%
IV	85	28.1%
Stage			
Early (I+II)	162	53.5%
Advanced Level (III+IV)	141	46.5%
	Mean (SD.)	Median (Min-Max)
CRP (mg/dL)	2.68 (3.33)	0.70 (0.15-14.50)
Albumin (g/dL)	3.67 (1.36)	3.90 (1.00-6.60)
Lymphocytes (10^3^/µL)	2.53 (1.00)	2.50 (1.10-4.50)
CALLY index	1.78 (1.64)	2.24 (0.01-6.88)
CA 15-3 (U/mL)	53.18 (53.06)	41.00 (3.00-407.00)

SD, Standard Deviation. (Variables: CRP, Albumin, Lymphocyte count, CALLY index, CA 15-3; summarized as Mean ± SD and Median [Min-Max]).

CRP level was 0.45 mg/dL (0.30-0.50) in Stage I and 5.5 mg/dL (4.30-9.90) in Stage IV, and the increase was significant (p< 0.001). CRP level was similar in Stage I and II patients and showed a significant increase from Stage II onwards. Albumin level was 4.65 g/dL (4.00-5.20) in Stage I and 2.3 g/dL (1.90-3.30) in Stage IV. Albumin levels were similar in Stage I and II patients. Lymphocyte level decreased from 3.2 10³/μL (2.60-4.10) in Stage I to 1.4 (1.10-2.10) in Stage IV. The CALLY index showed a dramatic decrease from 3.42 (2.73-4.03) in Stage I to 0.06 (0.04-0.11) in Stage IV. Similarly, the CALLY index of Stage I and Stage II patients was similar (p= 0.582). CA 15–3 values were similar between Stages III and IV, but showed a significant increase up to Stage III (**Table 2**).

**Table 2:** Comparison of laboratory parameters according to individual clinical stages.

Stage		CRP (mg/dL)	Albumin (g/dL)	Lymphocytes (10^3^/µL)	CALLY index	CA 15-3 (U/mL)
		Median (Q1-Q3)	Median (Q1-Q3)	Median (Q1-Q3)	Median (Q1-Q3)	Median (Q1-Q3)
	I (n=62)	0.45 (0.30-0.50)	4.65 (4-5.20)	3.2 (2.60-4.10)	3.42 (2.73-4.03)	20.5 (11-32)
	II (n=100)	0.4 (0.30-0.58)	4.20 (3.70-5)	3 (2.50-3.50)	2.92 (2.54-3.39)	34 (19-55)
	III (n=56)	2.5 (2.10-3.20)	3 (1.90-4.10)	2 (1.30-2.75)	0.2 (0.14-0.30)	60.5 (32-87)
	IV (n=85)	5.5 (4.30-9.90)	2.3 (1.90-3.30)	1.4 (1.10-2.10)	0.06 (0.04-0.11)	65 (41-90)
p Value		<0.001	<0.001	<0.001	<0.001	<0.001
Pairwise Comparisons	I vs II	0.999	0.317	0.999	0.582	0.002
	I vs III	<0.001	<0.001	<0.001	<0.001	<0.001
	I vs IV	<0.001	<0.001	<0.001	<0.001	<0.001
	II vs III	<0.001	<0.001	<0.001	<0.001	<0.001
	II vs IV	<0.001	<0.001	<0.001	<0.001	<0.001
	III vs IV	0.001	0.618	0.154	0.001	0.999

Kruskal Wallis Test (Monte Carlo), *Post Hoc* Test: Dunn’s Test, Q1, 1st Quarter; Q3, 3rd Quarter. (Statistical test: Kruskal-Wallis with Dunn’s *post hoc*; variables are shown as Median [Q1-Q3] for each stage I-IV).

Similarly, CRP levels were 0.4 mg/dL (0.30-0.50) in the early stages and 4.1 mg/dL (2.70-7.10) in the late stages and the difference was significant (p< 0.001). Similarly, albumin and lymphocyte levels decreased significantly in advanced stages (p< 0.001). CALLY index decreased from 3.08 (2.58-3.60) in early stages to 0.12 (0.05-0.19) in late stages (p<0.001). CA 15–3 levels were significantly higher in advanced stages (29 vs. 62 U/mL, p<0.001) (**Table 3**).

**Table 3:** Comparison of biomarkers between early (I+II) and advanced (III+IV) stages.

Biomarkers	Phase 2		p
	Early (n=162)	Advanced level (n=141)	
	Median (Q1-Q3)	Median (Q1-Q3)	
CRP (mg/dL)	0.4 (0.30-0.50)	4.1 (2.70-7.10)	<0.001
Albumin (g/dL)	4.4 (3.80-5.10)	2.7 (1.90-3.90)	<0.001
Lymphocytes (10^3^/µL)	3.1 (2.50-3.70)	1.7 (1.20-2.20)	<0.001
CALLY index	3.08 (2.58-3.60)	0.12 (0.05-0.19)	<0.001
CA 15-3 (U/mL)	29 (15-45)	62 (37-89)	<0.001

Mann Whitney U Test (Monte Carlo), Q1, 1st Quarter; Q3, 3rd Quarter. (Statistical test: Mann-Whitney U with Monte Carlo simulation).

**Figure 1:**
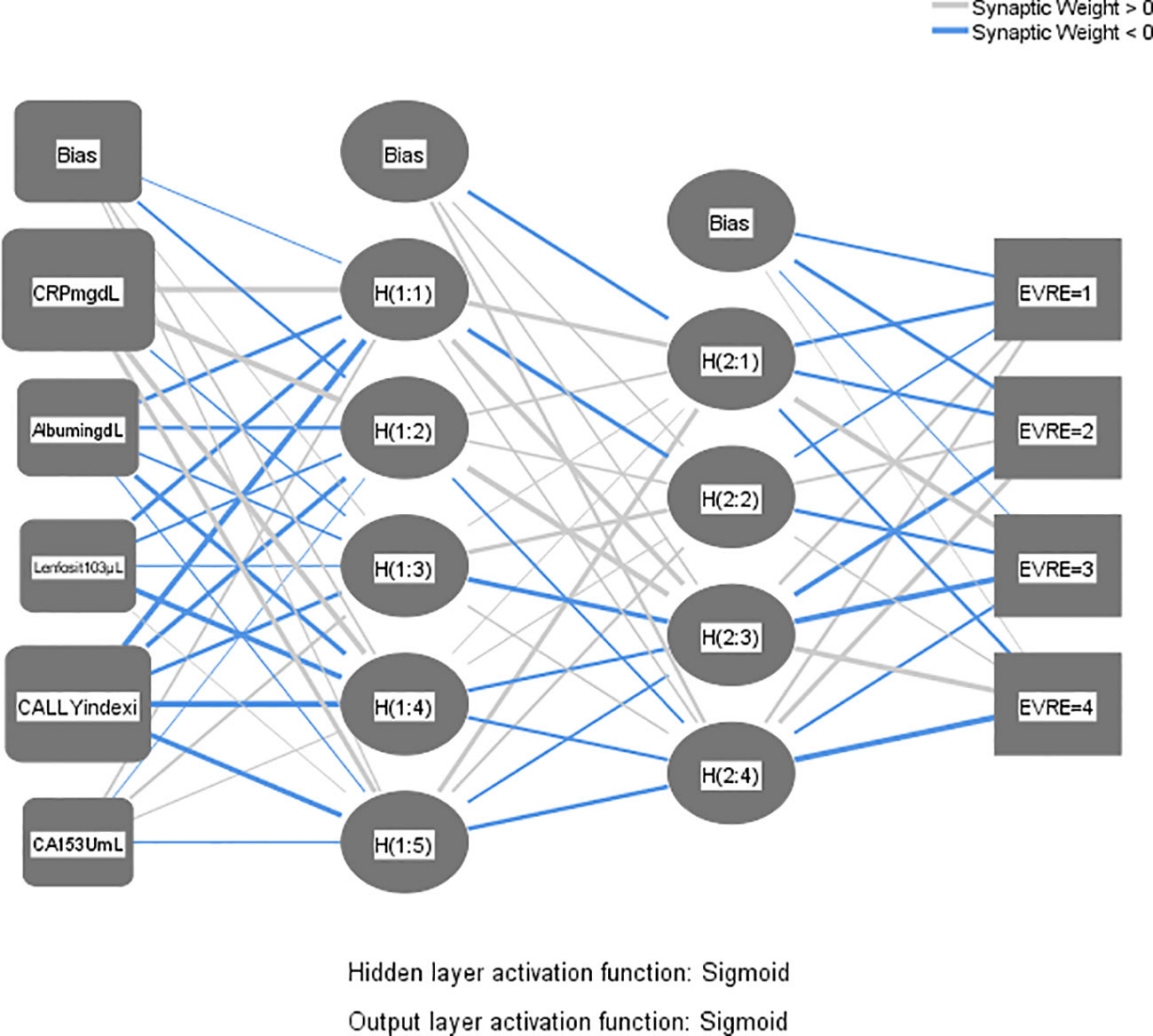
Stage prediction accuracy with confusion matrix or neural network (training set).

**Figure 2:**
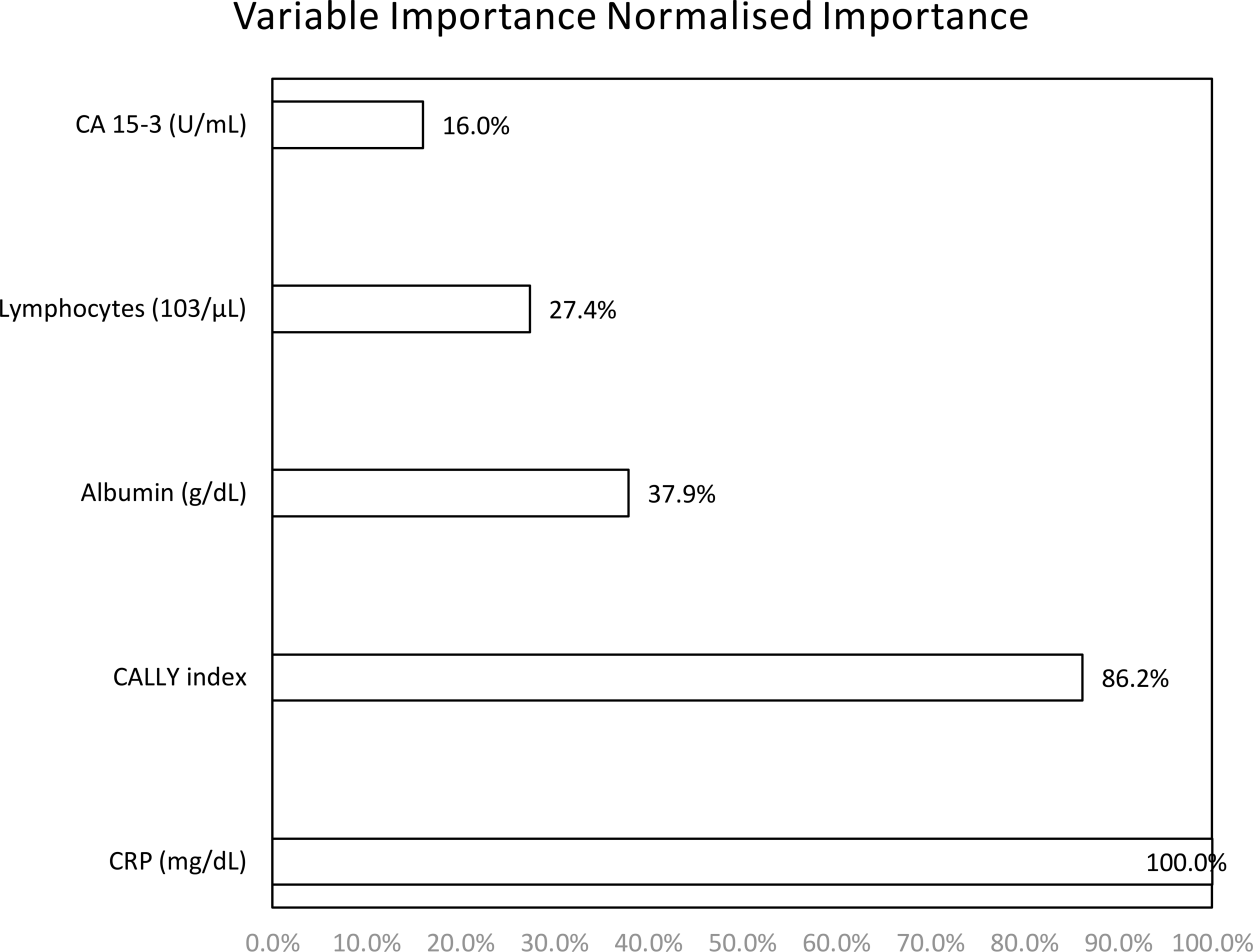
Variable importance graph from neural network classifier. (CRP, CALLY index, albumin, lymphocyte count, showing the relative importance of CA 15-3).

According to the Neural Network and Variable Importance analysis, the predictive power of the variables was as follows; CRP (Normalized Importance: 1.000), CALLY index (0.862), albumin (0.379), lymphocyte count (0.274) and CA 15-3 (0.160), (Max. importance: 1.000). The accuracy rates of the model were calculated as 80.2% in the training set and 84.9% in the test set. Especially stage II-IV patients were classified with high accuracy (**Table 4**, **Figures 1**, **2**).

**Table 4:** Predictive importance of variables in neural network classifier for staging.

Variable importance		Sample (hold)	Estimated				
Independent variable	Normalized importance		I	II	III	IV	Correct percentage
CRP (mg/dL)	100.0%	Education (70%)					
CALLY index	86.2%	I	16	27	0	0	37.2%
		II	5	65	0	0	92.9%
		III	0	0	35	3	92.1%
		IV	0	0	8	58	87.9%
Albumin (g/dL)	37.9%	General Percentage	9.7%	42.4%	19.8%	28.1%	80.2%
Lymphocytes (10^3^/µL)	27.4%	Test (30%)					
		I	9	9	1	0	47.4%
CA 15-3 (U/mL)	16.0%	II	1	29	0	0	96.7%
		III	0	0	17	1	94.4%
		IV	0	0	1	18	94.7%
		General Percentage	11.6%	44.2%	22.1%	22.1%	84.9%

Neural Network (Multilayer Perceptron), Hidden layer activation function: Sigmoid, Output layer activation function: Sigmoid, Dependent Variable: Phase (I-II-III-IV). (Algorithm: multilayer perceptron; normalized importance and classification accuracy by stage).

**Figure 3:**
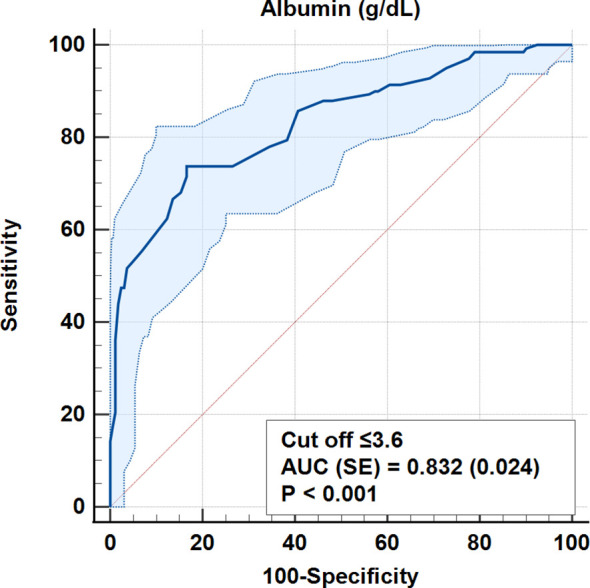
ROC curves for albumin in differentiating early and advanced stages.

**Figure 4:**
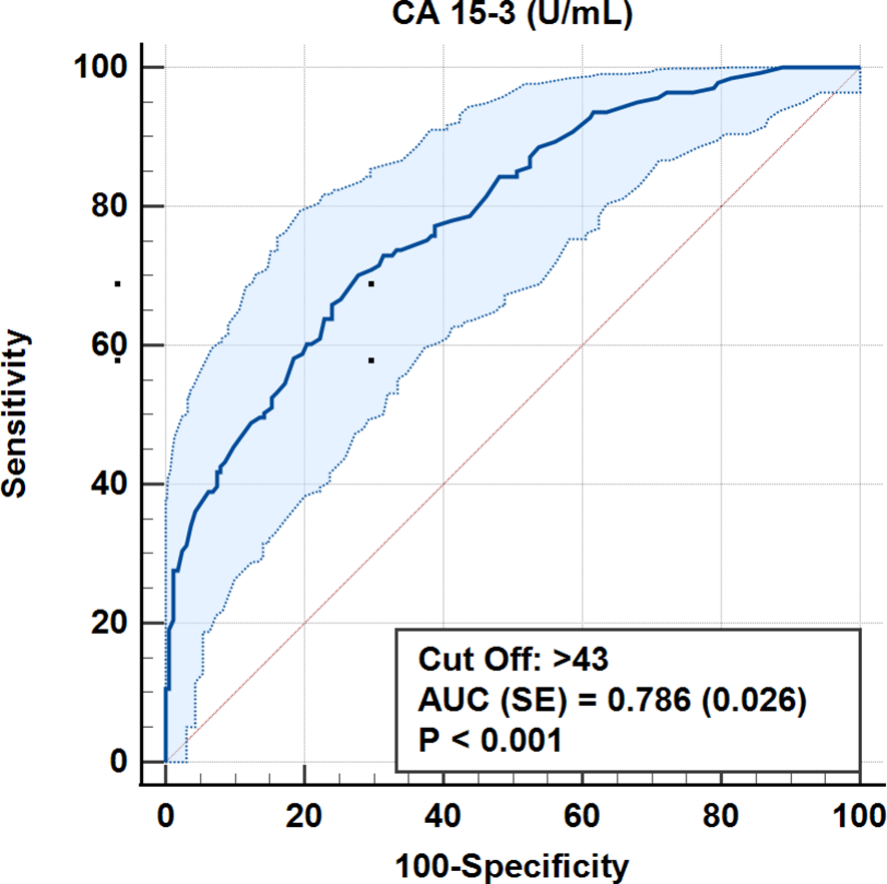
ROC curves for CA 15–3 in differentiating early and advanced stages.

**Figure 5:**
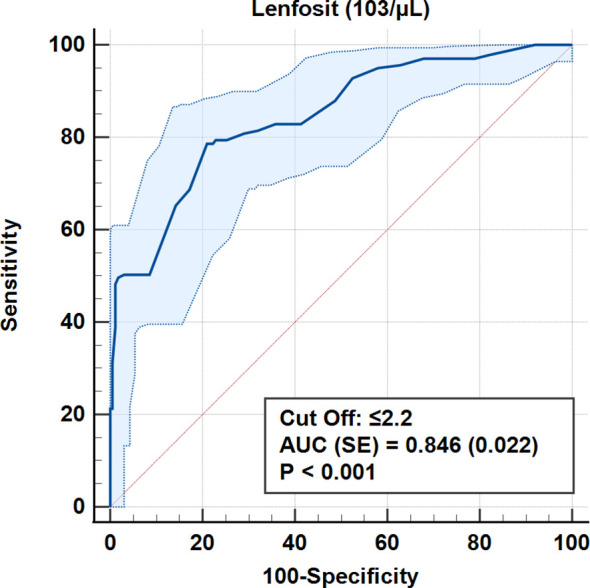
ROC curve for lymphocyte count in differentiating early and advanced stages.

**Figure 6:**
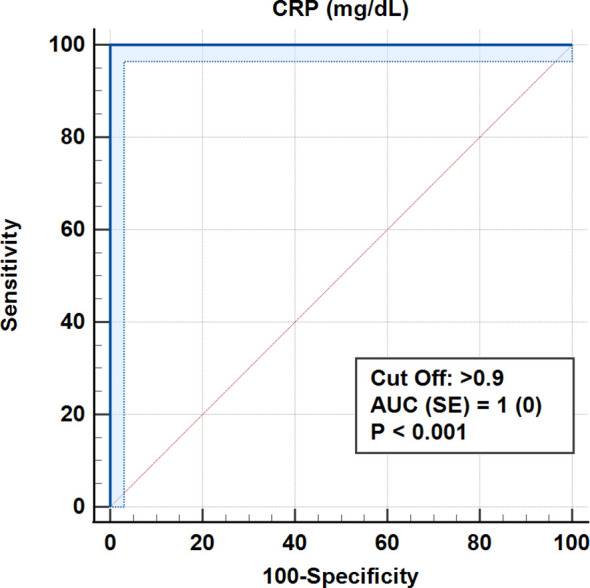
ROC curve for CRP in differentiating early and advanced stages.

**Figure 7:**
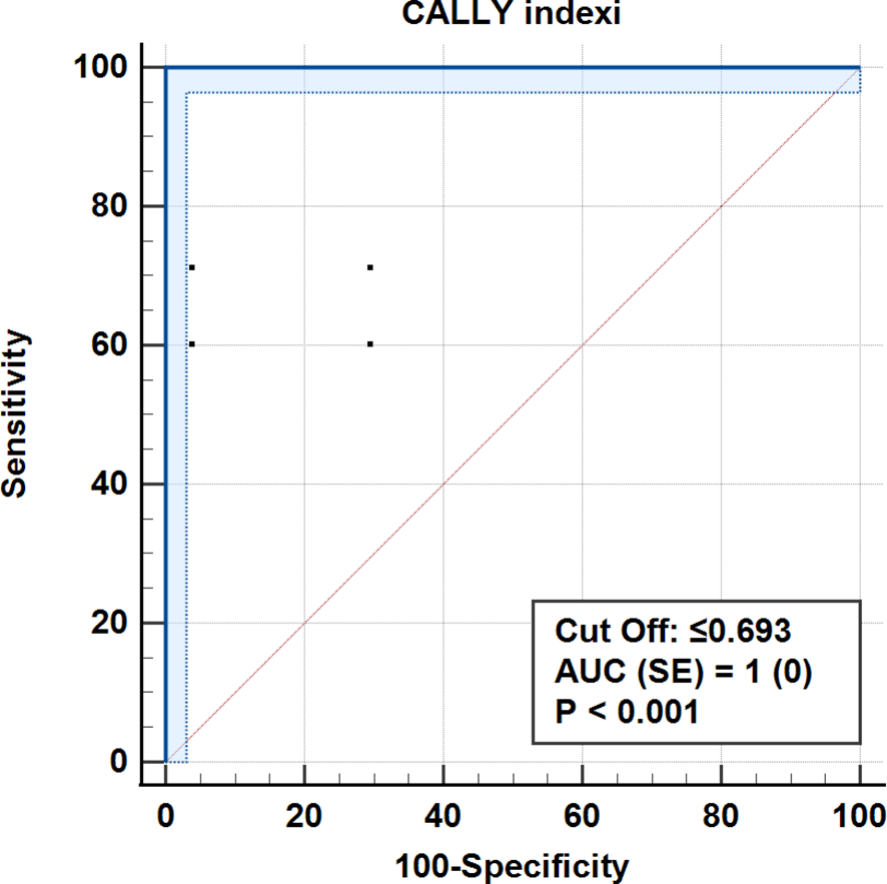
ROC curve for the CALLY index for distinguishing early from advanced stages.

According to ROC analysis, the accuracy for the cut-off value of 0.693 for the CALLY index was 100% (AUC = 1.0, p < 0.001). Similarly, for CRP values, 100% accuracy was obtained for a cut-off level of 0.9 mg/dL (AUC = 1.0, p < 0.001) (**Table 5**, **Figures 3**–**7**).

**Table 5:** Diagnostic performance of individual biomarkers in predicting clinical stage (early vs advanced), (ROC analysis, cutoff-based classification, AUC, sensitivity, specificity, precision, F1-score, MCC).

Biomarkers	Early (I+II)	Advanced level (III+IV)	Accuracy	sensitivity (PPV)	F1 Score	Matthews correlation coefficient (MCC)	AUC ± SE.	P
	n (%)	n (%)						
CALLY								
>0.693	162 (100.0)2s	0 (0.0)	100.00%	100.00%	1	1	1 ± 0	<0.001
≤0.693	0 (0.0)	141 (100,0)sp						
CRP								
≤0.9	162 (100.0)s	0 (0.0)	100.00%	100.00%	1	1	1 ± 0	<0.001
>0.9	0 (0.0)	141 (100,0)sp						
Lymphocyte								
>2.2	128 (79,0)s	30 (21.3)	78.90%	81.00%	0.8	0.58	0.846 ± 0.022	<0.001
≤2.2	34 (21.0)	111 (78.7)sp						
CA-153								
≤43	117 (72,2)s	42 (29.8)	71.30%	73.60%	0.73	0.42	0.786 ± 0.026	<0.001
>43	45 (27.8)	99 (70,2)sp						
Albumin								
>3.6	135 (83,3)s	37 (26.2)	78.90%	78.50%	0.81	0.57	0.832 ± 0.024	<0.001
≤3.6	27 (16.7)	104 (73.8)sp						

Roc (Receiver Operating Curve) Analysis (Honley & Mc Nell - Youden index J) AUC, Area under the ROC curve; SE, Standard Error; CI, Confidence interval; PPV, Positive predictive value; ss, Sensitivity; ssp, Sensitivity.

## Discussion

In studies using the CALLY index, lymphocyte count, serum albumin, and C-reactive protein (CRP) are assessed together to provide a comprehensive immune nutritional assessment. In a study conducted by Zhuang et al., the prognostic significance of the CRP-Albumin-Lymphocyte (CALLY) index was evaluated in 174 women with non-metastatic invasive breast cancer undergoing surgery (24). In this study, the CALLY index was examined in terms of overall survival and disease-free survival, and the optimal cut-off point for the CALLY index was determined as 2.285 using an ROC curve (Specificity: 72.4%, AUC: 0.730; Sensitivity: 71.7%). Higher CALLY index values ​​were found to be significantly associated with longer overall survival and disease-free survival. The CALLY index was found to outperform lymphocyte count, albumin, and CRP components in terms of prognostic accuracy. In this manuscript, the CALLY index was found to be significantly higher in patients with early-stage breast cancer. CALLY index decreased from 3.08 in early stages to 0.12 in late stages. According to ROC analysis, the accuracy for the cut-off value of 0.693 for the CALLY index.

A study by Zhu et al. reported that the CALLY index, shows significant prognostic value in stage III breast cancer. Patients with high CALLY values had longer survival time than those with low CALLY values (25).

Pathological complete response after neoadjuvant chemotherapy is a validated endpoint for long-term survival in HER2-positive breast cancer. Karatli et al. investigated the predictive role of the CALLY index in determining pathological complete response after neoadjuvant chemotherapy in HER2-positive breast cancer. They showed that high CALLY index and high Ki-67 were statistically significant in predicting pathological complete response (26).

CA15–3 is a serum tumor marker for breast cancer that is still widely used in clinical practice. Studies have shown that elevation of CA15–3 levels in patients with early breast cancer and normal serum CA15–3 level has a prognostic impact (6).

In this manuscript, analyzed the relationship between TNM stages and the CALLY index in predicting early and advanced breast cancer. Using traditional statistical methods and machine learning-based classification models, the diagnostic accuracy of the CALLY index was evaluated in comparison with the tumor marker CA 15-3. Unlike the CA 15–3 value, the CALLY index was found to be significantly higher in patients with early-stage breast cancer and significantly lower in patients with advanced breast cancer. The CALLY index has been shown to have diagnostic accuracy in breast cancer staging. Therefore, the CALLY index demonstrated discriminatory performance in predicting early-stage breast cancer. Furthermore, supervised machine learning models confirmed that the CALLY index was the variable with the highest normalized significance in stage classification. Among the evaluated models, the multilayer perceptron network achieved the highest prediction accuracy of 84.9%, reinforcing the clinical utility and reliability of the CALLY index in pretreatment staging.

### Power limitations

Several limitations should be considered when interpreting the results of this study. The retrospective nature of our study, its single-center nature, limited patient population, the limited diagnostic performance of CA 15-3, the lack of comparison with other inflammatory indices, and its status as the first study on this topic pose limitations for external validation. This limits the generalizability of the findings and may reduce the statistical power to detect subtle associations.

## Conclusion

The results of our study show that the CALLY index is one of the non-invasive and easily accessible biomarkers that can distinguish between early and advanced breast cancer by integrating inflammatory, nutrition and immune parameters. Furthermore, the CALLY index holds significant potential for pre-treatment prognostic stratification of breast cancer in clinical practice, but larger-scale studies are needed.

## Data Availability

The datasets presented in this study can be found in online repositories. The names of the repository/repositories and accession number(s) can be found in the article/supplementary material.
